# Intestinal Epithelial Cells-Derived Hypoxia-Inducible Factor-1α Is Essential for the Homeostasis of Intestinal Intraepithelial Lymphocytes

**DOI:** 10.3389/fimmu.2019.00806

**Published:** 2019-04-16

**Authors:** Lihua Sun, Teming Li, Hanlin Tang, Kun Yu, Yuanhang Ma, Min Yu, Yuan Qiu, Pengyuan Xu, Weidong Xiao, Hua Yang

**Affiliations:** ^1^Department of General Surgery, Xinqiao Hospital, Third Military Medical University, Chongqing, China; ^2^Department of Gastrointestinal Surgery, The Second Affiliated Hospital of Kunming Medical University, Kunming, China

**Keywords:** intestinal epithelial cells, hypoxia-inducible factor-1α, intestinal intraepithelial lymphocytes, inflammatory bowel disease, CD8

## Abstract

Hif-1α is a master regulator which involved in the transcriptional regulation of anti-inflammatory or cellular responding to hypoxia. Previous work shows that the absence of Hif-1α results in the destruction of intestinal epithelial cell (IEC) and abnormalities of intestinal barrier function. However, we know very little about other functions of Hif-1α on intestinal intraepithelial lymphocyte (IEL). Therefore, we generated a transgenic mouse (Hif1-α^*ΔIEC*^ mice), which was knocked out Hif1-α specifically in IECs, to study the effect of Hif1-α on IEL. IELs were isolated from the small intestine and colon of mice, respectively, and examined by flow cytometry and quantitative real-time PCR. All the cytokines expression was detected by quantitative real-time PCR. The NSAID enteropathy was induced by gavaged with 5 mg/kg indomethacin and the experimental colitis was induced by administration of 2.5% DSS. We found that the number of IELs is increased in Hif1-α ^*ΔIEC*^ mice. It is showed that knockout of Hif1-α in IECs led to significant changes in IEL phenotype, including a marked decline in the CD8αα^+^ and TCRγδ^+^ population. The reduction of CD8αα^+^ IELs is accompanied by increased apoptosis, decreased proliferation and weakened migration in Hif1-α^*ΔIEC*^ mice. Moreover, absence of intestinal epithelial Hif1-α markedly changed the population of IELs in NSAID-induced small intestinal injury and increased susceptibility to dextran sulfate sodium-induced colitis. In summary, our results first time demonstrate that IEC-derived Hif1-α is essential for maintaining IELs homeostasis and intestinal microbiota.

## Introduction

Intestinal immune homeostasis depends on tightly regulated crosstalk among commensal bacteria, mucosal immune cells, and intestinal epithelial cells (IECs). Disruption of this homeostasis leads to an inflammatory disorder of the gastrointestinal tract (1). Previous studies demonstrated that IEC-derived regulatory signals controlled innate and adaptive immune-cell function in the intestinal microenvironment. Disruption of IEC-intrinsic pathways results in dysregulated innate and adaptive immune responses in mouse models of inflammatory bowel disease (IBD) (2). According to reports, IECs regulate dendritic cells (DCs) function through the secretion of immunoregulatory molecules including thymic stromal lymphopoietin (TSLP), transforming growth factor-β (TGF-β), and prostaglandin E_2_ (2–4). It is reported that IEC-derived TSLP can also influence mast-cells activation (2, 5). In addition to effects on mast-cells and DCs, there is increasing evidence that IEC-derived signals can positively and negatively regulate T and B cell functions in the intestine (2, 6). In contrast to the crosstalk between IECs and these immune cells, the influence of IECs on intraepithelial lymphocytes (IELs) is much less clear.

IELs are a distinct population of T lymphocytes, which are located at the basolateral side of IECs in the epithelial layer of the intestine (7). IELs play an important role in maintaining intestinal homeostasis and defense against intestinal pathogens (8). Previous studies of small intestinal IELs showed that IELs could possess several features that distinguish them from peripheral lymphocytes in structure, function, origin, development and homing (9, 10). Traditionarily, IELs can be classified into two major subgroups based on their differential expression of T cell receptors (TCR): TCRαβ^+^ cells and TCRγδ^+^ cells (11). However, IELs can also be divided into another two groups based on their expression of co-receptor molecules: “Type A or induced IELs” and “Type B or natural IELs.” Type A IELs include conventional CD4^+^ and CD8αβ^+^ T cells; Type B IELs include unconventional CD8αα^+^ T cells (12). In mice, about 65–75% of small intestinal IELs are “Type B IELs” (13).

Our and others previous studies demonstrated that IEC played an important role in regulating IELs phenotype and function by secreting various kinds of cytokines (7, 14). For example, epithelial-derived IL-7 has particular importance in the intestines as an extrathymic regulator of γδ IEL differentiation and growth (7, 15, 16). Mice lacking IL-7 showed severely decreased γδ IEL numbers (15). Oppositely, mice with IEC-specific over-expression of IL-7 resulted in a significant expansion of the IEL population (7). Mice lacking IL-15 also shows a severe reduction in CD8αα^+^ TCRαβ^+^ and TCRγδ^+^ IELs (17). It shows that IL-18 produced by IECs, in collaboration with other cytokines such as IL-7 and IL-15, plays a crucial role in the proliferation and development of intestinal IELs (18). One latest research shows that mice lacking IEC-intrinsic histone deacetylase 3 (HDAC3) results in CD8α^+^ TCRαβ^+^ IELs impaired function by IL-18 pathway during an enteric infection (19). However, how the IEL instructed by surrounding IEC is still remained obscure.

Hypoxia-inducible factor-1(HIF-1) is a heterodimeric transcription factor which involved in the transcriptional regulation of anti-inflammatory or tissue-protective signaling pathways (20–23). HIF-1 is composed of two subunits, the HIF-1α and HIF-1β subunits (24). As a key regulator of oxygen homeostasis, HIF-1α is tightly regulated by the level of oxygen. In contrast, HIF-1β is constitutively expressed (24, 25). Several studies have indicated that HIF-1α activation and function are central in intestinal epithelial cells (26–28). Karhausen et al. showed that loss of epithelial HIF-1α led to the abnormalities of intestinal barrier function and more severe colitis (26). Sun et al. found that IEC- derived HIF-1α contributed to the maintenance of mucosal homeostasis by inducing IL-33 expression in inflamed mucosa of IBD (28). Cummins et al. reported that HIF-1α inhibited epithelial cell apoptosis by suppression of HIF-1α degradation in murine colitis (27). Recent studies demonstrated that HIF-1α also played an important role in the regulatory T cells (Tregs) (29, 30). It is reported that HIF-1α in Dendritic cells (DCs) is necessary for the increased number of Tregs and induction of Treg-activating by the crosstalk between Tregs and DCs (29).

However, there is relatively little information about the role of epithelial HIF-1α in the regulation of IELs. Thus, the purpose of this study is to investigate the effects of IEC-derived HIF-1α deficiency on IELs homeostasis, functions, and intestinal inflammation.

## Materials and Methods

### Ethics Statement

All experiments were performed under specific pathogen-free conditions in conformity with ethical guidelines and approved by the Animal Ethics Committee of the Third Military Medical University.

### Mice

HIF-1α Flox mice (Hif1-α^*F*/*F*^ mice) with C57BL/6J background were kindly provided by Lin Chen (Research Institute of Surgery, Daping Hospital, Third Military Medical University, Chongqing, China). The Villin-Cre mice with C57BL/6J background were purchased from Shanghai Research Center for Model Organisms (Shanghai, China). Hif1-α^*F*/*F*^ mice were first crossed with Villin-Cre mice to generate heterozygous Villin-Cre- Hif1-α^*F*/+^ mice. Then, Villin-Cre- Hif1-α^*F*/+^ mice were backcrossed with Hif1-α^*F*/*F*^ mice to obtain VIL-Cre-Hif1α^*F*/*F*^, IEC-specific Hif1-α-deficient mice (hereinafter referred to as Hif1-α^*ΔIEC*^ mice) (31). Sibling mice negative for cre recombinase (Hif1-α^*F*/*F*^) served as controls (Wild Type, WT). All mice were housed and maintained in laminar flow cabinets under specific pathogen-free conditions. In order to determine the efficiency of gene knock-out, we isolated epithelial cells from small intestine and colon, respectively, and used q-PCR analysis of epithelial mRNA. The following primers were applied: Hif1-α forward, 5′-GTCCCAGCTACGAAGTTACAGC-3′; Hif1-α reverse, 5′-CAGTGCAGGATACACAAGGTTT-3′. All were performed under the guidelines of the Institutional Animal Care and Use Committee of Third Military Medical University.

### DSS Colitis Model

Male mice 6–8 weeks old were given 2.5% DSS (MP Biomedicals, Cleveland, USA) dissolved in drinking water for 7 days and body weight was recorded daily. On day 7, mice were killed by cervical dislocation, and colonic tissues were collected for stained with H&E and Flow Cytometric analysis.

### NSAID Enteropathy Model

Male mice (8 weeks old with a mean weight of 21–25 g) were gavaged once daily with indomethacin (5 mg/kg; for 7 days; Selleckchem, Houston, TX, USA) dissolved in DMSO and further diluted in phosphate buffered saline (PBS). On day 7, mice were killed by cervical dislocation, and the small intestine were collected for stained with H&E and Flow Cytometric analysis.

### IEC and IEL Isolation

Intestinal IEL isolation was performed according to the protocol as described previously by our laboratory (13, 32). Mesenteric fat and Peyer's patches were carefully removed. The small intestine or colon was opened longitudinally, washed in RPMI 1640 Medium, and cut into 5 mm pieces. Then, the pieces were incubated in extraction buffer (5 mM EDTA, 2 mM DTT, 10% fetal bovine serum in PBS) with continuous brisk stirring at 37°C for 30 min. The tissue suspension was filtered rapidly through a 40-μm strainer to remove debris and centrifuged at 2,000 rpm at 4°C for 5 min. Pelleted cells were suspended in 40%/70% isotonic Percoll (Sigma, USA) and centrifuged at 2,000 RPM at room temperature for 20 min. The supernatants contained an enriched IEC population. The IECs layer was carefully sucked off and captured for RNA extraction. IELs are at the 40–70% interface. The cells were washed twice and resuspended in RPMI 1640. The viability of the IELs exceeded 95%, according to trypan blue exclusion staining.

### Flow Cytometric Analysis

The number of IELs was calculated with CountBright™ absolute counting beads according to the manufacturer's instructions (Invitrogen). The IELs were stained with a combination of the following fluorescence-conjugated mAbs: CD3-PE-CY7, CD8α-APC, CD8β-FITC, CD4-PE, TCRβ-APC, TCRγδ-FITC, CD103-PE, and CD69-FITC. All antibodies were purchased from Biolegend (SanDiego, USA). Cells were suspended in 100 μL staining buffer (eBioscience, SanDiego, USA) with saturating amounts of antibodies and incubated for 30 min on ice and placed in the dark. The apoptotic ratios for the IELs were measured by flow cytometry according to the manufacturer's protocol. Flow cytometry was performed with standard techniques. The image acquisition and data analysis were performed with MoFlow (Beckman Coulter, US) and Flowjo (Three Star, Ashland, USA).

### BrdU Incorporation and Measurement

For BrdU pulse-chase experiments, BrdU (2 mg/mouse) was injected i.p. 12 h prior to analysis as previously described (17); isolated cells were stained with fluorescent antibodies specific for CD3, CD8α, and CD8β and conjugated BrdU antibody set (BD, USA) according to manufacturer's instructions and analyzed immediately by flow cytometry.

### Histological Examination

The excised intestinal tissues were fixed in 4 % paraformaldehyde for histological evaluation. The segments were embedded in paraffin and cut into 5 μm sections for staining with H&E. The histological evaluation was assessed under a light microscope (Leica, Germany) at × 200 magnification.

### Real-Time Quantitative PCR

Total RNA was extracted from isolated IELs or IECs using TRIzol (Invitrogen, Carlsbad, CA) following the manufacturer's instructions. Briefly, RNA was reverse-transcribed into complementary DNA (cDNA) with a SuperScript First-Strand Synthesis System RT-PCR kit (TaKaRa Bio Inc, Japan) and served as a template for amplification of IL-2, KGF, IFN-γ, RegIIIγ, IL-7, IL-15, IL-6, IL-10, and β-actin. The PCR reaction was performed with GoTaq® qPCR Master Mix (Promega, USA) on the Rotor-Gene Q PCR detection system (Qiagen, Germany). The primer sequences are as follows: KGF, F: TGGGCACTATATCTCTAGCTTGC, R: GGGTGCGACAGAACAGTCT; IL-2, F: TGAGCAGGATGGAGAATTACAGG, R: GTCCAAGTTCATCTTCTAGGCAC; IFN-γ, F: TGGCTGTTTCTGGCTTGTTACT, R: TGACGCTTATGTTGTTGCTGA; RegIIIγ, F: TTCCTGTCCTCCATGATCAAAA, R: CATCCACCTCTGTTGGGTTCA; IL-7, F: TTCCTCCACTGATCCTTGTTCT, R: AGCAGCTTCCTTTGTATCATCAC; IL-15, F: ACATCCATCTCGTGCTACTTGT, R: GCCTCTGTTTTAGGGAGACCT; IL-6, F: TAGTCCTTCCTACCCCAATTTCC, R: TTGGTCCTTAGCCACTCCTTC; IL-10, F: ACAGCCGGGAAGACAATAAC, R: CAGCTGGTCCTTTGTTTGAA; β-actin, F: AGCCATGTACGTAGCCATCC, R: TTTGATGTCACGCACGATTT.

### Enzyme Linked Immunosorbent Assay (ELISA)

The IECs were isolated from the small intestine of mice. After five cycles of freezing and thawing, proteins were released into the supernatant. Then, the protein concentration of IL-6, IL-10, IL-7, and IL-15 were determined using a specific ELISA kit (Mlbio, Shanghai, China) following the manufacturer's protocol.

### Gut Microbiota Analysis

Fecal pellets were collected from mouse colon and stored at −80°C. Bacterial DNA was extracted using the TIANamp Stool DNA Kit (TIANGEN, Beijing, China). The DNA samples were sent to proceed DNA sequencing with Illumina Miseq 2 × 300 at Shanghai Sangon Biotec Co., Ltd (Sangon, China).

### Statistical Analysis

All experimental data shown as the Mean ± SD. Statistical significance was determined by unpaired two-tailed Student *t* test or two-way ANOVA with Bonferroni *post hoc* analysis using GraphPad Prism version 7.0 software (San Diego, CA). *P* < 0.05 was considered statistically significant.

## Results

### Hif1-α Signaling Modulates the IECs Cytokines Expression

**Figure 1:**
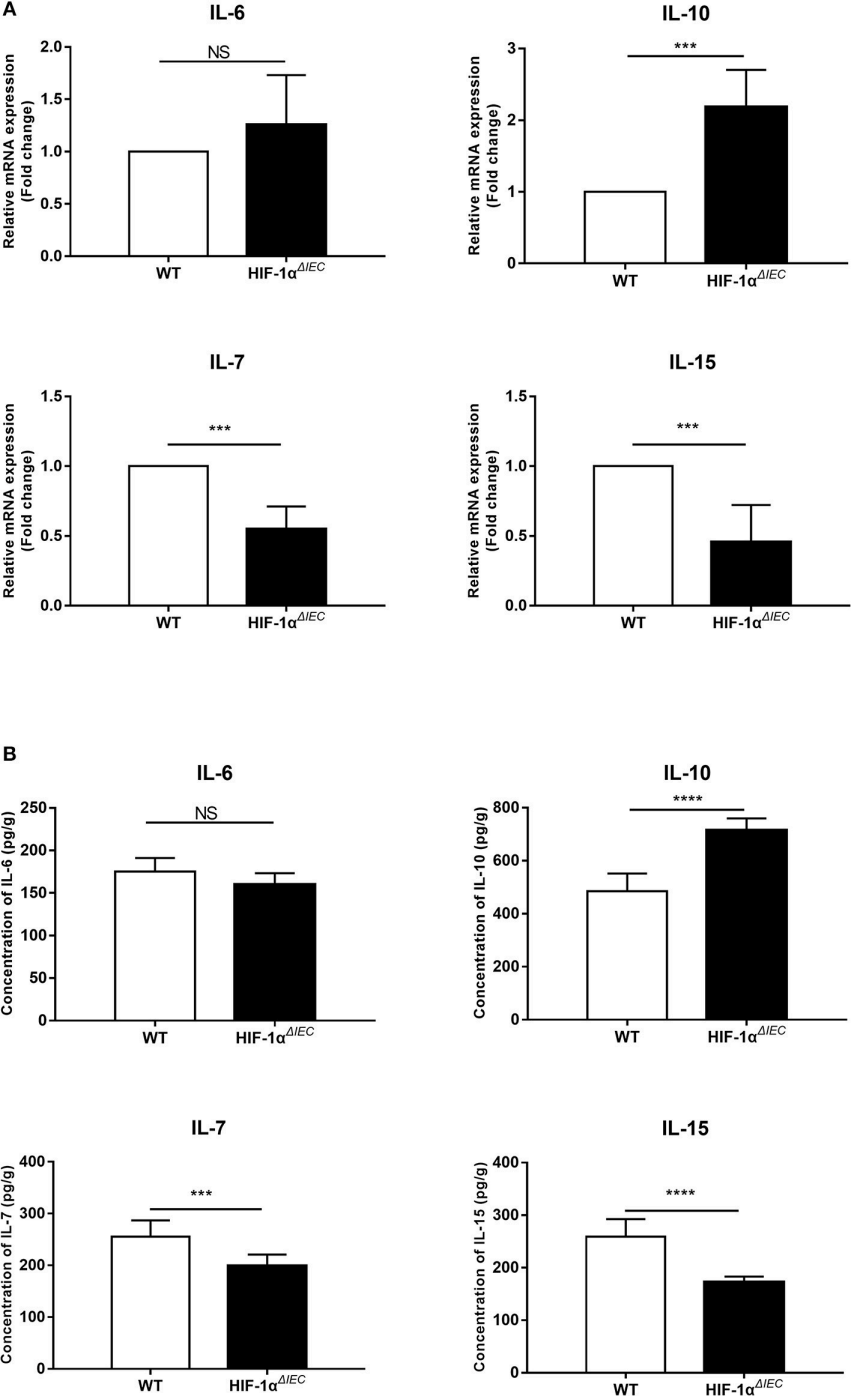
Hif1-α signaling modulates the intestinal epithelial cells (IECs) cytokines expression. mRNA **(A)** and protein **(B)** expression of IL-6, IL-10, IL-17, and IL-15 were examined in small intestinal epithelium samples from WT or Hif1-α^*ΔIEC*^ mice (*n* = 5–8 mice/group). ^***^P < 0.001; ^****^P < 0.0001; NS, not significant.

To study the effect of Hif1-α deletion on epithelial cells, we examined the expression of several cytokines mainly secreted by epithelial cells. IECs were isolated from the small intestine of WT and Hif1-α^*ΔIEC*^ mice, respectively. Real-time PCR analysis of the indicated cytokines is shown in Figure 1A. There is no significant difference in IL-6 expression between WT and Hif1-α^*ΔIEC*^ mice. The mRNA expression of IL-10 increased more than double in Hif1-α^*ΔIEC*^ mice. However, IL-7 and IL-15 in Hif1-α^*ΔIEC*^ mice showed a significant decrease in mRNA expression (about 54.88 and 45.67%, respectively) compared to the WT mice. Consistent with the Real-time PCR result, the protein expression measured by ELISA were shown in Figure 1B. We and others have previously reported that IL-7 and IL-15 were closely related to the development of IELs (33). Therefore, we speculated that Hif1-α deletion in IECs may affect the development of IELs. Then, we next examined the number, phenotype and function in IELs.

### Conditional Deletion of Hif1-α in Epithelia Results in the Increase of IELs Numbers in Small Intestine

**Figure 2:**
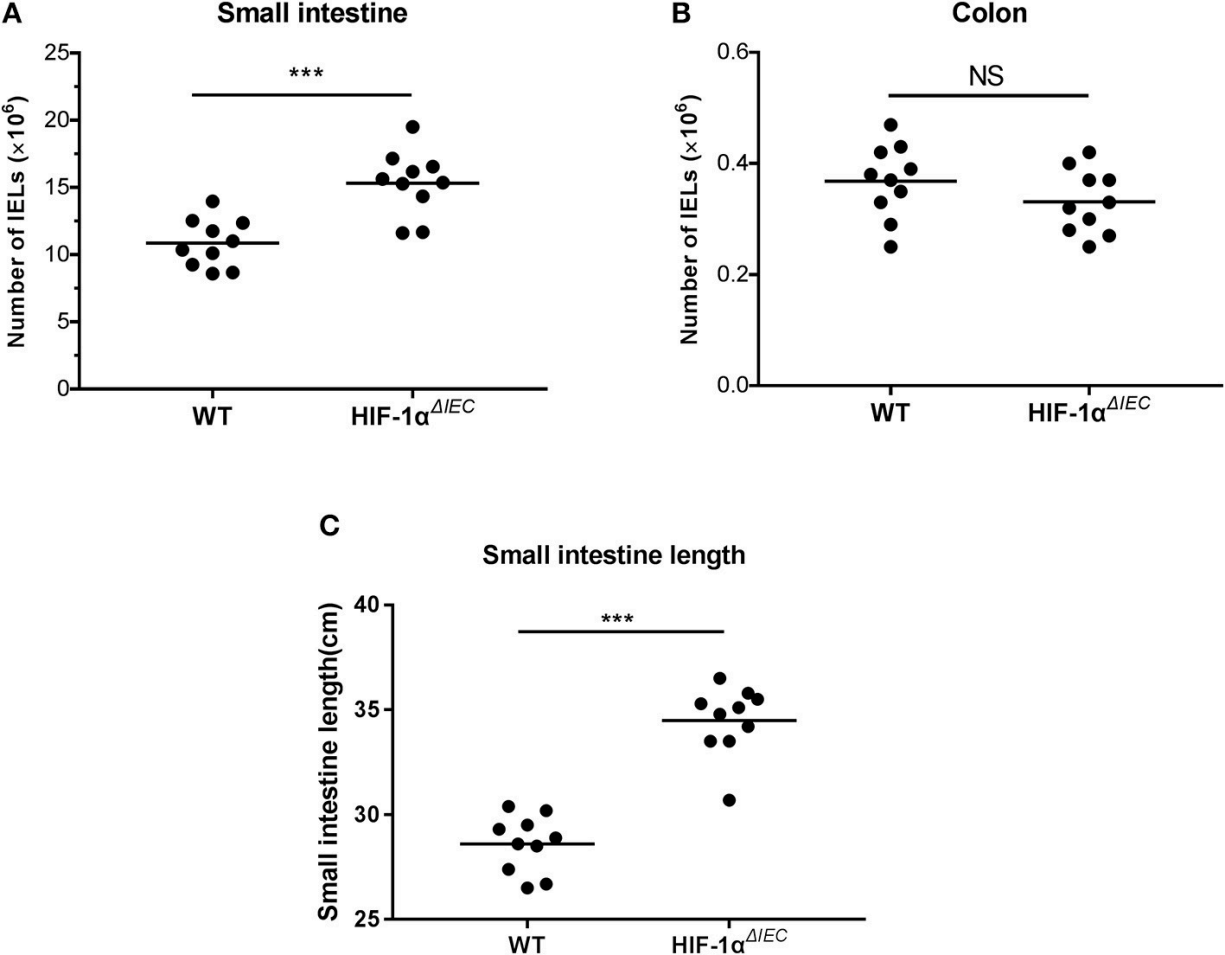
Changes in the number of intraepithelial lymphocytes (IELs) in Hif1-α^*ΔIEC*^ mice. The numbers of IELs in the small intestine **(A)** and colon **(B)** of Hif1-α^*ΔIEC*^ mice and individual control mice. **(C)** The length of small intestine from WT or Hif1-α^*ΔIEC*^ mice (*n* = 10 mice/group). ^***^P < 0.001; NS, not significant.

The number of IELs was accounted with CountBright™ absolute counting beads by flowcytometry. Among multiple samples, the total number of CD3^+^ cell increased about 1.5-fold (*P* = 0.0013) in the Hif1-α^*ΔIEC*^ mice compared to the WT mice in small intestine (Figure 2A). Conversely, there was a slight decrease (*P* = 0.0627) in the number of CD3^+^ cells in the Hif1-α^*ΔIEC*^ mice compared to WT mice in colon (Figure 2B). However, the decrease was not significant, for increased cell numbers could be due to an increase in length of the small intestine (duodenum, jejunum, and ileum). In our study, we found that the length of small intestine in Hif1-α^*ΔIEC*^ mice is apparently longer than that in WT mice (respectively, 34.49 ± 0.52 cm vs. 28.6 ± 0.43 cm) (Figure 2C). Therefore, this result showed that IEC-specific Hif1-α-deficient mice increased the total number of IELs and the length of small intestine.

### Conditional Deletion of Hif1-α in Epithelia Changes the Homeostasis of IELs

**Figure 3:**
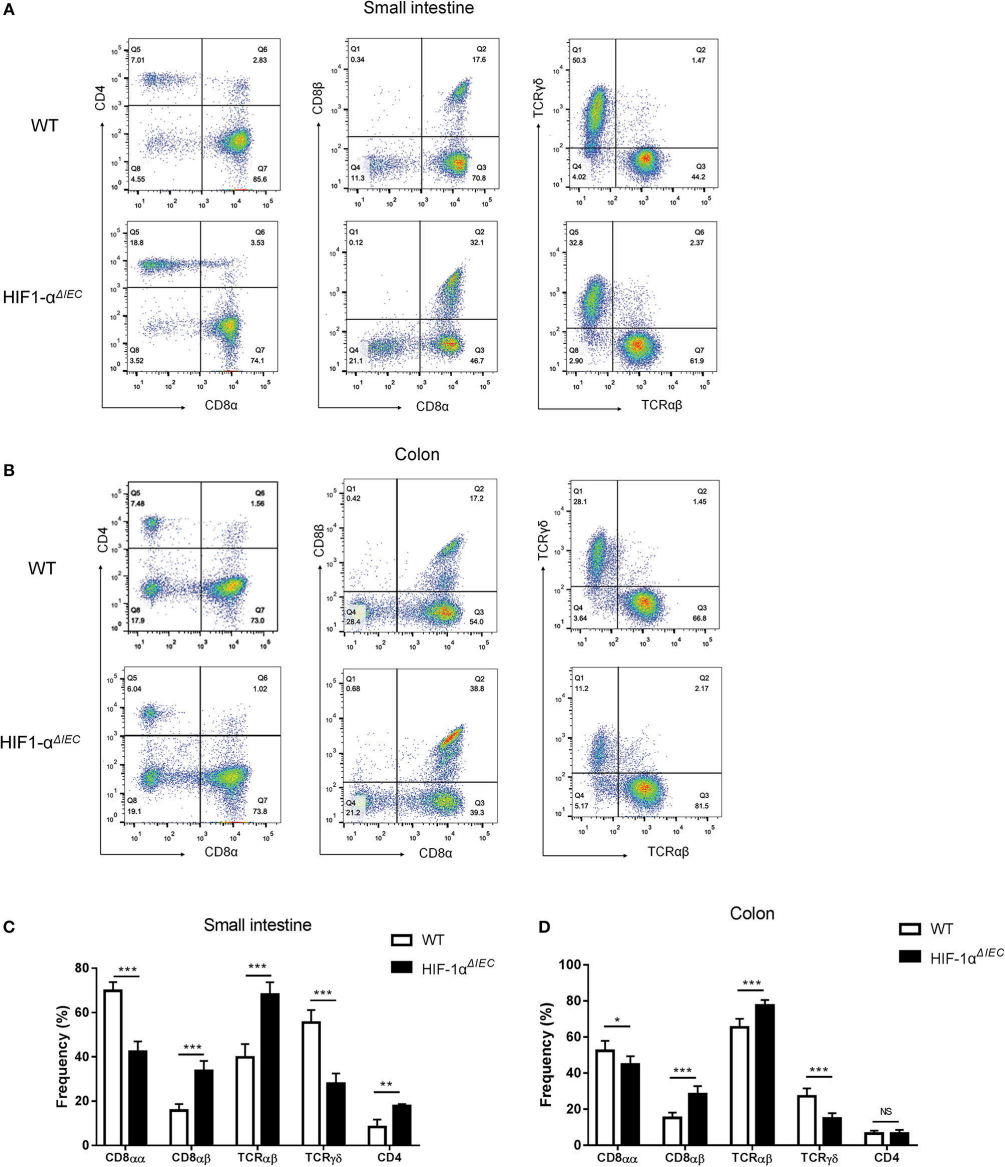
Changes in the phenotypes of intraepithelial lymphocyte (IEL) in Hif1-α^*ΔIEC*^ mice. Cell populations are expressed as the percentage of gated cells with different cell phenotype markers. The data were obtained fromCD3-positive cells. The IELs from small intestine **(A)** or colon **(B)** of mice were stained as indicated. Expression of CD4, CD8α, and CD8β chains on CD3^+^ IELs. Cells were stained with anti-CD3, anti-CD4, anti-CD8α, and anti-CD8β mAbs and positively gated by CD3. Expression of TCRαβ and TCRβ on CD3^+^ IELs. Cells were stained with anti-CD3, anti-CD8α, anti-TCRαβ, and anti-TCRβ mAbs and positively gated by CD3. The proportionality of the indicated IEL subsets in the small intestine **(C)** or colon **(D)** of individual mice (*n* = 5–7 mice/group). ^*^P < 0.05; ^**^P < 0.01; ^***^P < 0.001; NS, not significant.

To further explore the impact of epithelial Hif-1α knockout on the homeostasis IELs, we studied the phenotype of IELs by using flow cytometry. As shown in the Figure 3, the percentage of CD8αα^+^ IELs was dramatically reduced in Hif1-α^*ΔIEC*^ mice compared with WT mice both in the small intestine and colon (respectively, 69.83 vs. 42.29%, 52.33 vs. 44.78%). Correspondingly, CD8αβ^+^ IELs were increased significantly (respectively, 15.77 vs. 33.64%, 15.17 vs. 28.28%). Similarly, TCRγδ^+^ IELs in Hif1-α^*ΔIEC*^ mice were also obviously reduced in the small intestine and colon (respectively, 55.48 vs. 27.87%, 27.16 vs. 14.88%). However, there is a significant increase in the percentage of CD4^+^ IELs in Hif1-α^*ΔIEC*^ mice compared with WT mice in the small intestine, but not in the colon. The proportionality of IEL subsets are summarized in Figures 3C,D. Together, these results demonstrated that IEC- derived Hif-1α significantly influences the homeostasis of IELs, especially CD8α^+^ IELs.

### Destruction of IELs Homeostasis in Hif1-α*^*ΔIEC*^* Mice Is Due to the Functional Changes of CD8^+^ IELs

**Figure 4:**
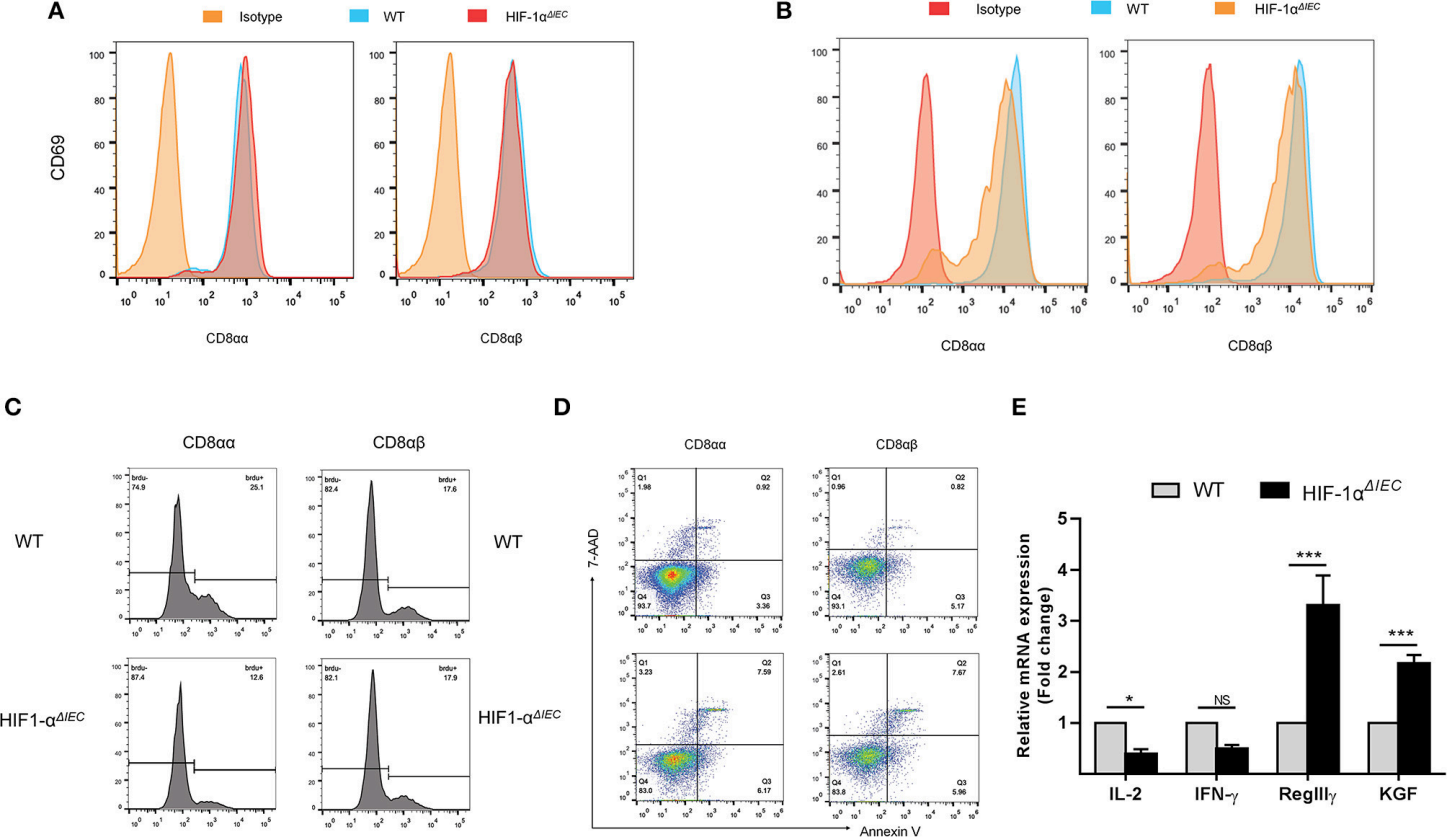
Destruction of IELs homeostasis in Hif1-α^*ΔIEC*^ mice is due to the functional changes of CD8αα^+^ IELs. The surface expression of CD69 **(A)** and CD103 **(B)** on CD8αα^+^ and CD8αβ^+^ IELs were detected by flow cytometry. **(C)** WT and Hif1-α^*ΔIEC*^ mice were injected with BrdU (2 mg/mouse). After 12 h, BrdU incorporation in the CD8αα^+^ and CD8αβ^+^ IELs were analyzed, respectively, by flow cytometry. **(D)** CD8αα^+^ and CD8αβ^+^ IELs were examined with apoptosis markers (Annexin V and 7-AAD) by flow cytometry. **(E)** Changes in the small intestinal IEL-derived cytokine mRNA measured using real-time RT-PCR. Data normalized to β-actin. (*n* = 4–7 mice/group). ^*^P < 0.05; ^***^P < 0.001; NS, not significant.

It is generally believed that CD8α^+^ T cells have regulatory properties and contribute to the maintenance of intestinal homeostasis (34). To further investigate the reasons for great changes in CD8α^+^ IELs caused by epithelial Hif-1α knockout, we assessed their proliferation, activation and apoptosis in the small intestine. CD69 is the early T cell activation marker, which can reflect the activated nature of IELs (32). So, we first detected CD69 expression by flow cytometry. The results showed no difference in the expression of CD69 both on the CD8αα^+^ and CD8α*β*^+^ IELs whether in WT or Hif1-α^*ΔIEC*^ mice (Figure 4A). We next examined the proliferative capacity of the two type IELs in small intestine by incorporation of BrdU *in vivo*. It is shown that CD8αα^+^ IELs present an extremely poor proliferation in Hif1-α^*ΔIEC*^ mice compared with WT mice; while CD8α*β*^+^ IELs have not variety (Figure 4C). Further study with apoptosis analysis showed that a twice higher apoptosis in CD8αα^+^ IELs and CD8α*β*^+^ IELs in Hif1-α^*ΔIEC*^ mice (Figure 4D). The CD103 have been associated with gut homing and retention, thus we speculate it will also be affected. Really, a significant decrement was found in the expression of CD103 on the CD8αα^+^ IELs from Hif1-α^*ΔIEC*^ mice (Figure 4B).

In addition, the expression of several cytokines, markers for IEL activation and intestine protection, were measured by real-time PCR. As shown in Figure 4E, there is a significant reduction on IL-2 and IFN-γ expression in the IELs from Hif1-α^*ΔIEC*^ mice, while obvious increase on KGF and RegIIIγ expression. Taken together, these findings demonstrated that changes in CD8α^+^ IELs function may be a major mechanism in breaking the homeostasis of IELs in Hif1-α^*ΔIEC*^ mice.

### Hif-1α Deficiency in Epithelial Markedly Changed the Population of IELs in NSAID-Induced Small Intestinal Injury

**Figure 5:**
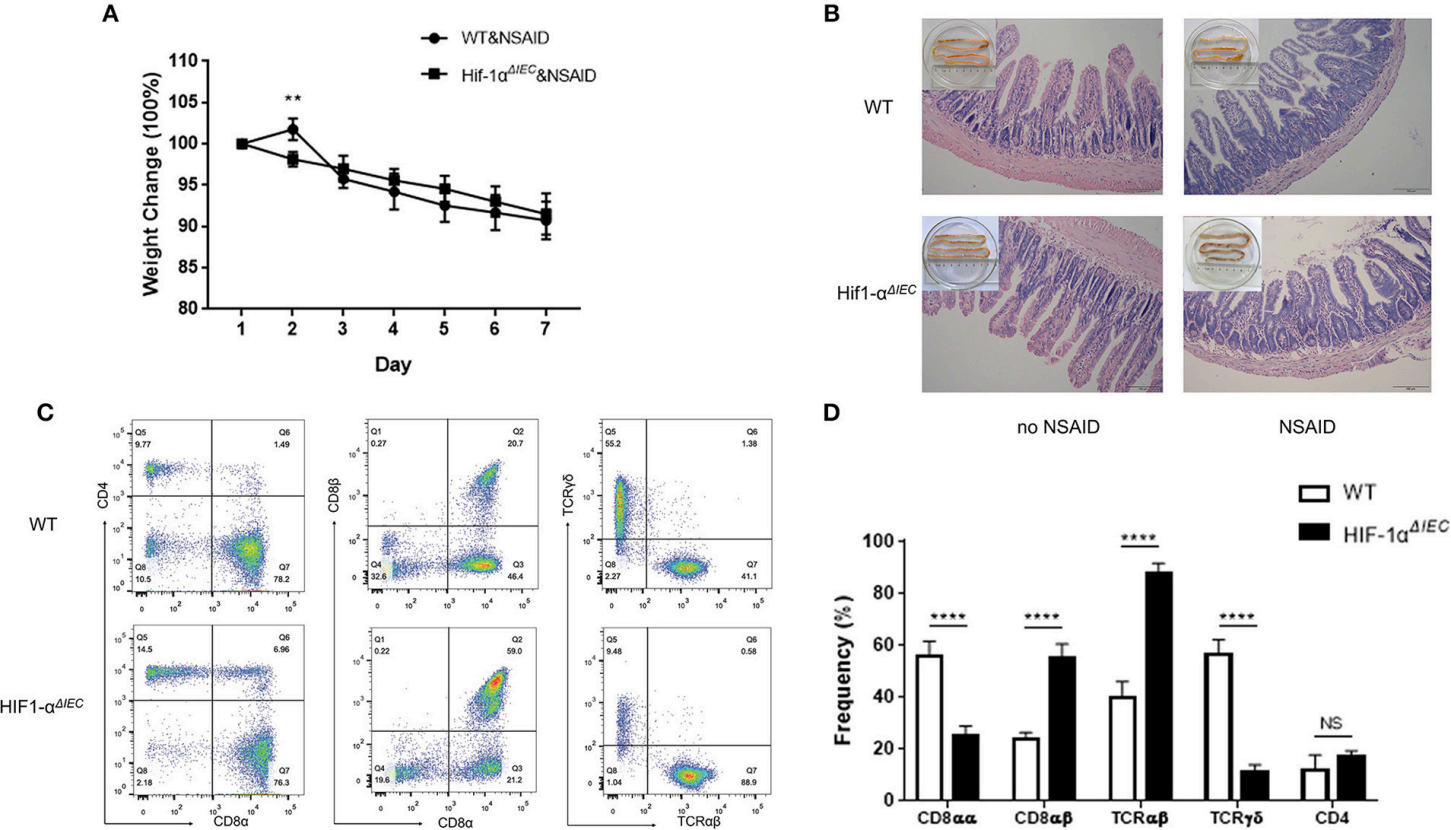
Hif-1α deficiency in epithelial markedly changed the population of IELs in NSAID-induced small intestinal injury. Damages were assessed in indomethacin-treated WT and Hif1-α^*ΔIEC*^ mice by **(A)** weight loss, **(B)** histological analysis, **(C,D)** the proportionality of the indicated IEL subsets in indomethacin-induced enteropathy (*n* = 5–7 mice/group). Images are 200x. ^**^P < 0.01; ^****^P < 0.0001; NS, not significant.

Non-steroidal anti-inflammatory drugs (NSAIDs) are associated with a high incidence of disorders in digestive tract mucosa (35). Because NSAIDs mainly causes damage to the small intestine, we investigated the effects of epithelial Hif-1α knockout in NSAIDs-induced small intestinal injury (enteropathy). We used indomethacin to gavage mice for 7 days and weighted every mouse every day. As shown in Figure 5A, the weight of all mice showed a gradual decline, except for the WT mice that increased slightly on the second day; besides, there is no difference between WT and Hif1-α^*ΔIEC*^ mice. Similarly, the indomethacin caused a thicker and shorter small intestine both on WT and Hif1-α^*ΔIEC*^ mice (Figure 5B). We also found that indomethacin did not produce noticeable macroscopic injuries whether in the intestinal tract of WT or in Hif1-α^*ΔIEC*^ mice by histological analysis (Figure 5B). The next study with IELs populations in mice by using flow cytometry confirmed the importance of IECs-derived Hif1-α in the development of NSAID-induced enteropathy. The proportion of CD8αα^+^ IELs was significantly decreased about 50% in Hif1-α^*ΔIEC*^ mice compared to WT mice, while CD8αβ^+^ IELs was significantly increased more than twice (Figure 5C). Strikingly, the proportion of TCRγδ^+^ IELs in Hif1-α^*ΔIEC*^ mice was vigorously reduced by 80% (Figure 5C). The proportionality of IEL subsets in NSAID-induced enteropathy were summarized in Figure 5D.

### Hif-1α Deficiency in Epithelial Aggravate DSS-Induced Colitis Severity

**Figure 6:**
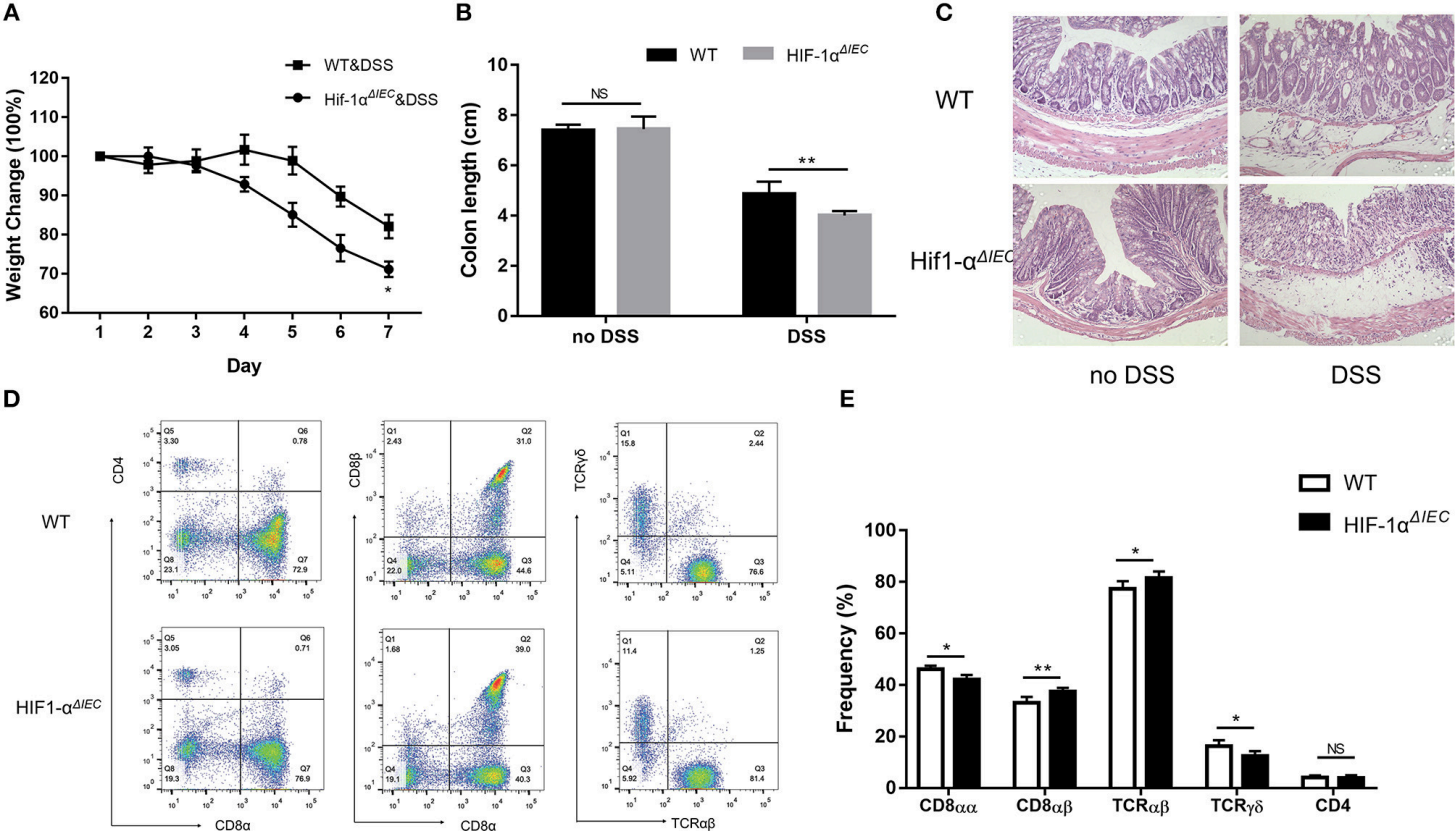
Hif-1α deficiency in epithelial aggravate colitis severity. Inflammation was assessed in DSS-treated WT and Hif1-α^*ΔIEC*^ mice by **(A)** weight loss, **(B)** colon length, **(C)** colon histological damage. **(D,E)** the proportionality of the indicated colon IEL subsets in DSS-induced colitis (*n* = 5–7 mice/group). Images are 200x. ^*^P < 0.05; ^**^P < 0.01; NS, not significant.

The destruction of IELs homeostasis in mice may aggravate colitis in several animal models and result in impaired ability to repair damaged epithelium (13). Therefore, we used 2.5% DSS-induced colitis model to address the consequences of IEC- derived Hif-1α deficiency for intestinal physiology. As shown in Figure 6A, both DSS-treated groups induced diarrheal bloody stools and experienced progressive weight loss begin on the 4th day. However, Hif1-α^*ΔIEC*^ mice lost more weight than WT mice; the difference in weight loss was statistically significant at days 6 and 7 (Figure 6A). Also, the colon length of Hif1-α^*ΔIEC*^ mice were shorter than WT mice in DSS-induced inflammation (Figure 6B). Again, the histological examination of colonic tissues confirmed that DSS-treated Hif1-α^*ΔIEC*^ mice showed more injury than WT mice (Figure 6C). However, no difference was observed between WT mice and Hif1-α^*ΔIEC*^ mice without DSS-treating. Further study with IELs subpopulations in Hif1-α^*ΔIEC*^ mice by using flow cytometry also confirmed the importance of Hif1-α in the development of DSS-induced colitis. As shown in Figure 6D, the proportion of CD8αα^+^ IELs was slightly decreased in Hif1-α^*ΔIEC*^ mice compared to WT mice. Similarly, the proportion of TCRγδ^+^ IELs in Hif1-α^*ΔIEC*^ mice was also slightly reduced. The proportionality of IEL subsets in DSS-induced colitis were summarized in Figure 6E.

**Figure 7:**
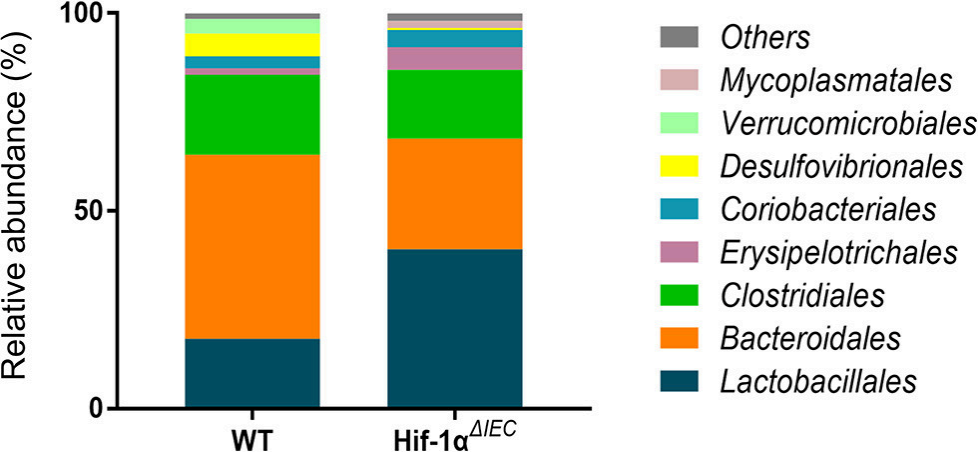
Changes in the relative abundance of several bacterial phyla in Hif1-α^*ΔIEC*^ mice. Bacterial community of fecal samples from WT and Hif1-α^*ΔIEC*^ mice with DNA sequencing (*n* = 6 mice/group).

Because Hif1-α also play important roles in host mucosal defense and response to microbial pathogens (36). Then, we examined the population of major microbial species by fecal DNA sequencing. It is shown that bacterial abundance is significantly changed in Hif1-α^*ΔIEC*^ mice compared with the WT mice without any treatment; demonstrating increased Erysipelotrichales, Lactobacillales and decreased Bacteroidales, Desulfovibrionales (Figure 7). The raw sequences of this study have been deposited in the Sequence Read Archive (accession number SRP166048). Taken together, these findings demonstrate that Hif-1α signaling influences the colon sensitivity to inflammation and establishment of the gut microbiome.

## Discussion

In this study, we first found that IEC-specific deletion of Hif1-α resulted in a significant decrease of the IL-7 and IL-15 mRNA and protein expressions in IECs. IL-7 and IL-15 are generally accepted as capital cytokines which involved in regulating the development and propagation of IELs (14, 18). So, we speculate that the loss of Hif1-α in IECs may affect the function of IELs. Next, our data provide the first evidence demonstrating that IEC-specific Hif1-α-deficient mice have undergone tremendous changes in IEL number, population, phenotype, proliferation, apoptosis, cytokine expressions, major microbial species, and inflammatory stimulation, compared to WT mice.

Interactions between IELs and IECs are thought to be crucial for maintaining intestinal mucosal immunity. Previous studies by our group have shown that IELs play an important role in the maintenance of the gut barrier function (37). Also, both experimental and clinical studies demonstrate that IECs may have an important role in mucosal immune responses by positively and negatively regulating IEL numbers and homeostasis. IECs can produce IL-6, IL-10, and other important cytokines for the stimulation and development of IELs. It has also been shown that IECs act as antigen presenting cells for both CD4^+^ and CD8^+^ IELs (7, 14). Recently, a study from our laboratory showed that TLR2 deficiency contributes to the impaired innate immune defense and high susceptibility to colitis in mice by changing IELs (13). These changes include a loss of the number of IEL populations and CD8αα^+^ IELs. BecauseTLR2 is only expressed in IECs in the mouse small intestine. So, TLR2^−/−^ mice is actually equivalent to IEC-specific deletion of TLR2 gene. This research once again proved to us that the influences of IEC-derived signaling pathways on the development of IELs.

Hif1-α is regarded as a master regulator of the cellular response to hypoxia and a crucial heterodimeric transcription factor (38). In our previous studies, we focused on the effects of Hif1-α on the intestinal epithelial barrier and colorectal cancer (39–41). However, more and more studies showed that Hif1-α develops a key role in the regulation of many immune cells in recent years. Dang et al. reported that HIF-1α controls the balance between Tregs and Th17 differentiation (42). Cramer and Walmsley et al. reported that HIF-1α promotes cell survival and migration, invasiveness, and bacterial killing in myeloid cells (29, 43). As with myeloid cells, DCs are also regulated by HIF-1α in cell differentiation, migration and survival (44). Therefore, we explored the effect of Hif1-α signaling pathway from IECs on IELs.

Our result found that the total number of IELs in Hif1-α^*ΔIEC*^ mice was increased significantly in the small intestine compared with WT mice. As increased cell numbers could be due to an increase in length of the small intestines. As expected, we found that the length of small intestines in Hif1-α^*ΔIEC*^ mice is apparently longer than in WT mice, but not in colon. It is reported that global deficiency of Hif1-α resulted in a developmental arrest at E9.0 and die by E10.5 (45, 46). These studies proved that Hif1-α plays an important role in embryonic development and survival. Therefore, this may be one of the reasons why the small intestine of Hif1-α^*ΔIEC*^ mice longer than WT mice. To further investigate the influences of IEC-derived Hif-1α in directing IEL lineage, we studied the phenotype of IELs. We found that the proportion of IELs subtypes has changed dramatically. It is shown that CD8αα^+^ IELs decreased significantly while CD8α*β*^+^ IELs increased accordingly. Through detection of function, we believe that CD8αα^+^ cells decrease may be due to the increased apoptosis, reduced proliferation and attenuated migration. Similar to our findings, a recent paper by Asis et al. noted that Hif1-α deletion in T cells have also significantly reduce the ratio of CD8^+^ T cells in tumor-infiltrating lymphocytes (TILs) by controlling effector cell differentiation, migration, and function (47). However, the function of CD8α*β*^+^ IELs were not changed except for apoptosis. Therefore, we speculate that the increase in CD8α*β*^+^ IELs may only be due to the decrease in CD8αα^+^ IELs.

NSAIDs are among the most frequently used medications worldwide for routine relief of pain or fever, to manage various forms of arthritis and inflammatory intestinal disorders, and to prevent or treat alimentary cancers (48). Recently, it is reported that the prevalence of NSAIDs-induced small intestinal damage was >50% in chronic users (49). Thus, it is meaningful to understand the pathophysiology of NSAIDs-induced enteropathy. Previous studies reported that Hif1-α expression augments inflammation in the proximal colon of sulindac-treated mice (31). However, few researchers study the effect of Hif1-α on small intestinal lesions caused by NSAIDs. In our researches, we found that indomethacin did not produce noticeable macroscopic injuries whether in the intestinal tract of WT or in Hif1-α^*ΔIEC*^ mice. However, the IELs homeostasis have changed dramatically between WT and Hif1-α^*ΔIEC*^ mice after treatment of indomethacin. Previous studies have shown that the deficiency in TCRγδ^+^ IELs in mice leads to an increase in the sensitivity of the small intestine to indomethacin-induced injury (50). We found that TCRγδ^+^ IELs in Hif1-α^*ΔIEC*^ mice was vigorously reduced in indomethacin-induced intestinal injury. This is indicated that the absence of Hif1-α in IECs aggravated NSAIDs-induced enteropathy, although there is no difference in histological analysis.

IBD is manifested by chronic inflammation of the gastrointestinal tract with significant morbidity and at times, life-threatening complications (51). Current studies suggest that the pathogenesis is complex and diverse. Previous studies reported that the absence of Hif1-α in IECs has aggravated inflammation in IBD (26). In addition, CD8αα T cells also are regulatory cells that suppress IBD symptoms in the gastrointestinal tract (52). In our researches, the change of CD8αα^+^ IELs percentage between Hif1-α^*ΔIEC*^ and WT mice is not obvious in DSS-induced colitis compared to no DSS treated mice. It may be that the CD8αα^+^ IELs have already present significant reduction in Hif1-α^*ΔIEC*^ mice compared to WT mice under physiological conditions, which also laid a hidden danger for the induction of DSS. Because the reduction of CD8αα^+^ IELs leads to the susceptibility of mice to enteritis. So, the CD8αα^+^ IELs in Hif1-α^*ΔIEC*^ mice are reduced to a lesser extent when the enteritis breaks out. These researches are enough to confirm our results on DSS induced colitis. Furthermore, many studies report a link between Hif1-α and bacteria (36). So, we sought to investigate the specific impact of an intestinal epithelial Hif1-α deletion on intestinal microecology. Hif1-α^*ΔIEC*^ mice were found to have increased bacterial loads and a shift of species, compared to the WT mice, suggesting that microbial dysbiosis in Hif1-α^*ΔIEC*^ mice may sensitize the colonic mucosa to chemical injury induced by DSS. Future studies will focus on the fecal transplantation.

In conclusion, our data indicate that intestinal epithelium Hif1-α plays a fundamental role in IELs homeostasis through the functional change of CD8αα^+^ IELs on proliferation, apoptosis and immigration. Simultaneously, our study also suggests that IEC-derived Hif1-α may represent a master regulator of enteritis and intestinal microbiota. There are mutual interactions between Hif1-α, CD8αα^+^ IELs and the microbiome that still remain to be fully understood. Although the complex regulatory network still needs to be fully elucidated, our findings are important for a better understanding on the crosstalk between LECs and IELs through Hif1-α.

## Ethics Statement

All animal procedures were performed under the guidelines of the Institutional Animal Care and Use Committee of Third Military Medical University.

## Author Contributions

LS and TL contributed equally to this work and conceived the study and analyzed the data. HT, KY, YM, MY, and YQ performed the research. PX, WX, and HY wrote the manuscript.

### Conflict of Interest Statement

The authors declare that the research was conducted in the absence of any commercial or financial relationships that could be construed as a potential conflict of interest.
